# “The police came in white protective suits and with batons, it was pure disaster” – a multi-stakeholder perspective on infection control in reception centers for asylum seekers during the COVID-19 pandemic in Germany

**DOI:** 10.1186/s12889-024-19925-5

**Published:** 2024-09-09

**Authors:** Latife Pacolli-Tabaku, Amand Führer, Diana Wahidie, Ulrich Trohl, Yüce Yilmaz-Aslan, Patrick Brzoska

**Affiliations:** 1 Health Services Research Unit, Faculty of Health, School of Medicine, Witten/Herdecke University, Alfred-Herrhausen-Straße 50, 58448 Witten, Germany; 2 Institute for Medical Epidemiology, Biometrics and Informatics (IMEBI), Interdisciplinary Center for Health Sciences, Medical School of the Martin Luther-University Halle-Wittenberg, 06112 Halle, Germany

**Keywords:** COVID-19, Refugee shelters, COVID-19 impact, Mass quarantine, Germany, Refugees, Pandemic measures

## Abstract

**Background:**

The COVID-19 pandemic presented unprecedented challenges, particularly for vulnerable populations residing in confined settings such as refugee shelters: Physical distancing measures were challenging to implement in shelters due to shared rooms or communal use of kitchens and sanitary facilities, which increased the risk of infections. Meanwhile, individuals’ capabilities for individual protection strategies were severely impaired by the structure of the shelters. Consequently, shelters had the duty to develop and implement strategies for the prevention and handling of SARS-CoV-2 infections. The aim of this study was to explore the perspectives of refugees, NGO employees, and shelter directors regarding COVID-19-related measures in German refugee shelters. The study aimed to identify challenges and conflicts arising from implemented measures, as well as expectations for improved support during the pandemic.

**Methods:**

Semi-structured and narrative interviews were conducted with 6 refugees, 6 facility managers, 12 NGO staff, and 2 social service agency staff from February to August 2022. Qualitative content analysis was employed to analyze the data, identifying overarching themes and codes.

**Results:**

The study uncovered challenges and conflicts resulting from pandemic measures, particularly mass quarantine orders, within refugee shelters. Lack of transparency and ineffective communication worsened tensions, with refugees feeling distressed and anxious. The quarantine experience had a negative impact on refugees’ mental health, which was exacerbated by limited social interaction and leisure-time activities. Shelter managers encountered administrative challenges when implementing measures due to facility constraints and limited resources, while NGO employees encountered obstacles in providing immediate assistance due to legal regulations and a lack of cooperation from shelter managers.

**Conclusions:**

The study highlights that shelters are problematic institutions from a public health perspective. It shows the importance of implementing customized pandemic interventions in refugee shelters that take account of the diverse needs and experiences of both refugee and staff. To achieve this, we recommend to establish an ethics committee and involve various stakeholders in decision-making processes. Additionally, enhancing information dissemination to promote transparency and public understanding of measures is crucial. These insights can help develop comprehensive and effective pandemic plans for refugee shelters, ensuring better preparedness for future public health crises.

**Supplementary Information:**

The online version contains supplementary material available at 10.1186/s12889-024-19925-5.

## Background

In March 2020, the World Health Organization (WHO) declared a pandemic due to the rapid global spread of the novel coronavirus [1]. As a result, governments around the world implemented partly drastic measures of physical distancing, and later mandated the closure of businesses, schools, and higher education institutions.

During the early stages of the COVID-19 pandemic, facilities housing individuals in close quarters, such as nursing and retirement homes or refugee shelters, were identified as a significant epidemiological concern [3–5]. Despite warnings from public health stakeholders about the precarious situation in shelters for refugees, the debate in Germany remained centered on nursing and retirement homes [4, 6].

In Germany, accommodation settings vary by Federal state. However, asylum seekers are usually initially accommodated in a reception center. A common characteristic of these refugee shelters is a high population density, with a significant number of people living in confined spaces [7–9]. During the pandemic, these housing and living conditions, which had already been criticized in the past [7, 10, 11], posed a particular challenge. Notably, recommendations for specific pandemic interventions for shelters were lacking for several months, despite warnings about the increased risk of infection in these settings [12]. Despite proposed measures to reduce viral transmission, such as physical distancing, hygiene protocols, testing initiatives, and isolation of infected individuals and their contacts [9, 13], implementation of these measures in such settings proved to be particularly challenging. This was primarily due to the persistence of crowded conditions, shared living spaces, and communal use of kitchens and sanitation facilities. As a result, a significant risk of transmission would remain even if the recommended measures were adopted [5, 12].

In addition to the aforementioned recommended measures, some shelters were fenced off, mass quarantine was ordered for all refugees in the shelter, and police reinforcements were called in to help enforce the mass quarantine measures [14, 15]. Due to these measures and even stricter control, the residents’ already tightly regulated daily life became even more regulated and constrained than before the pandemic [9]. Mass quarantine was implemented despite public health experts criticizing such approaches and demonstrating that mass quarantines have no epidemiological benefit and conflict with human rights [5, 12].

Public health experts share a broad consent that policy makers implementing pandemic measures designed primarily to maintain physical health must also consider the psychological impact these measures may have [16, 17]. The isolation and quarantine measures can be an additional burden for refugees who already have higher than average levels of psychological distress due to traumatic experiences before or during their journey to seek refuge [18–21]. Furthermore, since these recommended measures focus on dealing with the pandemic and do not address potential strategies for mitigating the impact of measures such as collective quarantine, it is important to consider the role and impact of these measures on all groups within shelters, such as refugees, NGO employees and shelter directors [9, 12, 13, 18, 21]. Still, research that combines the perspectives and experiences of NGO workers, refugee shelter directors, and refugees regarding COVID-19 and its impact on this specific population has been rare. Further insights into these perspectives can contribute to a more nuanced understanding of the challenges faced by this vulnerable group and inform targeted interventions to support their well-being beyond the pandemic.

The present qualitative study aims to address this limitation and to contribute to the development of pandemic interventions that adequately address the diverse lived realities of individuals residing or working in these settings. We particularly aimed to explore how different stakeholders perceived the infection control measures implemented during the COVID-19 pandemic, identify most challenging situations and conflicts arising from the infection control measures taken, and examine the expectations and aspirations of different stakeholders in terms of improved support during the pandemic. By exploring these aspects, we aim to help ensure that future pandemic interventions are more effective and better adapted to the needs and experiences of these specific target groups.

## Methods

### Study design

This study is based on the qualitative part of a mixed-methods project that was conducted from January 2021 to December 2022 and that examined the implementation of COVID-19 measures in reception facilities for refugees. Details on the mixed-methods design and the results from the other parts of the project are reported elsewhere [13, 22].

### Participants and participant selection

In the course of the study, we conducted interviews with three distinct groups of stakeholders: those involved in the management of refugee shelters, representatives of nongovernmental organizations, and people who were living in shelters during the initial phases of the pandemic.

Participants were recruited using purposive sampling with the aim of representing all relevant groups of actors. For this purpose, we contacted the management of refugee shelters and representatives of nongovernmental organizations working in these settings and asked them to put us in contact with potential respondents that worked in refugee shelters in the early phase of the pandemic. In addition, we recruited participants through a survey that was conducted in a previous phase of the project. Refugees were recruited through nongovernmental organizations. We only included respondents who were at least 18 years of age and who provided their informed consent for participation.

### Data collection

The interviews were conducted from February to August 2022. We employed two types of interviews as the method of data collection: semi-structured interviews and narrative interviews [23]. For both types, the interview guide was created collectively by all authors based on existing research in the field (an English-language version of the interview guides can be found in the Supplement of this article). For the semi-structured interviews, it contained five sections: (1) measures for dealing with the pandemic; (2) facility-related challenges; (3) conflicts during the pandemic; (4) ethical and social aspects of the measures; and (5) respondents’ expectations for improved support during pandemic times. The narrative interviews opened with the question “How did you first feel the effects of the pandemic and how did it influence your life?” In cases where the interviewee’s narrative was brief or initially superficial, a series of follow-up questions were used to try to gain further insight.

Our choice of method followed established approaches in qualitative research and selected the type of interview based on the respective object of investigation [34]: To investigate the rather circumscribed professional experience of the experts (management of refugee shelters and representatives of nongovernmental organizations) in a time-efficient manner, we chose semi-structured interviews. In contrast, for a nuanced and comprehensive understanding of refugees’ experiences during the pandemic, narrative interviews that give more space to the subjective experience of the interviewed were deemed the right method of data collection.

Most interviews were conducted by the two first authors L.P.T. and A.F. via telephone or video call in either German or English depending on the preference of the interviewees. Three interviews were conducted in Dari by Dari-speaking author D.W. Interviews with female refugees were conducted by L.P.T., the other interviews were conducted by either L.P.T. or A.F. Overall, we interviewed 6 refugees using narrative interviews, and 6 facility managers, 12 NGO staff, 2 social service agency staff using semi-structured interviews. Interviewees were sampled until information saturation was reached and the collection of further data did not provide any new findings. In total, interviewees were recruited from 14 different shelters. The duration of the interviews ranged from 20 to 80 minutes.

### Data analysis

The interviews were audio-recorded. In addition, the interviewers took notes during the interview. Immediately after the interview, the authors L.P.T. and A.F. documented the interview using a pre-tested documentation sheet. In cases where we failed to recollect all needed details we took recourse to the audio recording. We hereby followed established procedures for the conduct of rapid ethnography [24, 25]. In the course of the written documentation, the interviews were anonymized.

We used qualitative content analysis according to Kuckartz to systematically analyze the material, utilizing the MAXQDA analysis software which enables a combination of both inductive and deductive procedures for a comprehensive analysis of the qualitative data [26, 27]. The deductive category system was created using the main themes of the interview guide. The resulting categories were expanded during the analysis and supplemented with inductive categories. The process of coding was performed by one author (L.P.T), and subsequently reviewed and validated by a second author (A.F.) for accuracy and potential revisions. During the analysis, the codes were compiled and discussed among all authors. Twenty-three codes were identified and organized into seven overarching themes.

In the next section, we present the themes and categories, and use representative quotes from the interviews as illustration.

## Results

In the following, we will outline the themes identified in the data and sketch the categories each theme comprised. Hereby, we will focus on four thematic areas that particularly illustrate the situation of refugees in camps during the COVID-19 pandemic. The themes are: challenges and conflicts encountered during the pandemic; the psychological well-being of the refugees; ethical considerations related to the implemented measures; and wishes and expectations for better support.

### Conflicts and challenges in the facilities during the COVID-19 pandemic

The implementation of mass quarantine orders within refugee shelters has resulted in significant conflicts, primarily attributed to a lack of transparency and ineffective communication. Refugees were confronted with unsettling circumstances when wire fences were hastily erected overnight without prior notice or information. Refugees reported feeling distressed and anxious due to the sudden and unexplained appearance of the wire fences, leaving them confused and unaware of the unfolding situation. The use of a megaphone to announce the quarantine measures exacerbated communication problems, creating difficulties for refugees with limited language skills to understand the information. The lack of clear communication increased tensions, resulting in resistance from refugees attempting to flee the perceived danger. According to the interviewees, in response to the resistance, both police and security personnel intervened with force. Dressed in white protective suits and armed with batons, their entry into the shelters was described as traumatic and considered marked by brutal violence that exacerbated existing conflicts. Refugees also reported a lack of information about the duration of and rationale for their enclosure, which severely constricted opportunities for social interaction and any leisure-time activities.

“When you are in [this camp], you are completely isolated, you are treated very badly, like you are in hell.” [Refugee, m].

According to NGO employees, it was understandable that refugees resisted mass quarantine due to a lack of information. During the initial stages of the quarantine, refugees were largely uninformed about the reasoning behind the measure, with only minimal communication. Despite the presence of police patrolling the quarantine facilities, supposedly no efforts were made to directly engage and inform the quarantined refugees. Throughout the mass quarantine period, NGO workers reported to have been denied access to the camp, preventing them from providing necessary information to refugees and from exercising their advisory role.

“The police came in white protective suits and with batons, it was a pure disaster” [NGO employee, m].

“Residents received no information about the mass quarantine. Instead, fences were simply erected, and the facility was monitored by private security services” [NGO employee, f].

### Refugees’ mental health situation

The quarantine experience caused significant stress for refugees, as for many it evoked memories of prior incarceration or mistreatment suffered in authoritarian transit or home countries. The constant presence of police officers tasked with monitoring and enforcing quarantine regulations exacerbated the situation. This made refugees feel criminalized and left them with limited options for improving their situation. One refugee even stated that the experience in the reception centers in Germany was worse than during their temporary stay in Greece, and that refugees were treated in an inhumane manner.

Simultaneously, other refugees reported symptoms such as a lack of motivation, fatigue, and bad mood during quarantine. Isolation and an uncertain future exacerbated these negative emotional states. The absence of opportunities for leisure-time activities and the fear of getting infected during mass quarantine left refugees with limited scope to improve their mood.

“It was not easy to see the police around you all the time, as if you were a criminal […] they watched you every second and they inspected and they controlled you” [Refugee, m].

“We were treated inhumanely, as if we were not human beings” [Refugee, m].

Meanwhile, refugee shelter managers faced significant administrative challenges, primarily revolving around implementing measures prescribed by public health authorities within tight time constraints. The structure of refugee shelters made it difficult to implement measures to reduce the spread of the COVID-19 pandemic in these shelters. The shelters’ housing of several hundred refugees, combined with physical constraints such as shared multi-bed rooms and communal use of kitchens and sanitary areas, added a layer of complexity to the implementation process. Directors had limited room for improvement under these conditions. They had to ensure that quarantine regulations were adhered to while also safeguarding the safety of the refugees. However, the implementation of these measures has had the opposite effect according to the interviewees, who felt that many of the measures supposedly designed to protect them actually increased the precarity of their situation.

“[We] arrived to work early and the area was fenced off” [Director, m].

Furthermore, the attitudes of refugee shelters‘ supervisory authorities were described as uncooperative. The sudden and unexpected enforcement of mass quarantine measures, as reported by the directors, introduced an additional layer of complexity to an already demanding process. The directors had limited time and ability to optimize the situation.

NGO employees also faced limited opportunities to improve the situation of refugees due to health authority regulations that restricted their access to shelters. This hindered their efforts and limited their ability to provide immediate assistance to residents. In some shelters, NGO employees encountered a lack of cooperation from managers, which made it difficult to implement improvements. This further constrained their scope for action and made it challenging for NGOs to offer support. The combination of governmental regulations and a lack of cooperation from managers resulted in limited capacity for NGO employees to enhance the situation of refugees in many cases.

### Ethical implications of the implemented measures

Participants were asked about their opinion on whether they felt the enforced measures violated fundamental rights or restricted the freedom of refugees. Most respondents stated that they did not perceive measures such as hand disinfection and mask wearing as a violation of fundamental rights, as they were implemented for the protection of refugees and staff and applied to the general population in Germany. However, the director of a refugee reception center acknowledged that these measures were not pleasant but justified as they served to protect the majority.

“You are ultimately paid to perform your function […] and also to enforce things that I don’t think are all that great, that I would perhaps rule differently, but where there is a certain necessity” [Director, m].

Restrictions on activities that structure the day, such as sports or kindergarten, were viewed more critically. All respondents considered measures such as mass quarantine, physical altercations or police intervention as a violation of fundamental rights and freedoms. Furthermore, a director of a shelter stated that refugees without daily structure and without opportunities for social interaction and any leisure-time activities easily fall into depression. Because of the measures implemented, difficulties emerged within the asylum process, which, according to the NGO employee, are also a violation of refugees’ fundamental rights. Refugees often faced challenges attending their scheduled asylum hearings during the quarantine period. Limited access to telephones made it difficult for some refugees to cancel or reschedule appointments. NGO staff also faced challenges in assisting refugees with canceling or rescheduling appointments due to limited access to shelters.

The following scenario as described by a NGO employee and a refugee in reference to the same shelter, illustrates the implementation of these measures in a facility: Following the discovery of three positive COVID-19 cases, approximately 300–400 refugees had collective quarantine measures imposed on them. The refugees not only faced the impact of the measures but also experienced a lack of individual notifications, which raised critical concerns about the transparency and fairness of the imposed restrictions. This case illustrates the practical consequences of the discussed opinions, providing a concrete example of the challenges faced by refugees when such measures were enforced. The shelter was secured immediately, with physical barriers and private security guarding the area. The interviewees considered these measures to be a violation of legal authority and an overstep of the law. This isolatated refugees from other parts of society, with their only remaining contacts being with government authorities. The perceived absence of protections against violence worsened the situation, resulting in the facility’s transformation into an institution where every aspect of life was predetermined and controlled. Furthermore, NGO employees stated that even prior to the pandemic, refugees’ rights and freedoms were not respected, and during the pandemic, decisions were taken without their consent. For example, members residing in the same household continued to be quarantined, even though the quarantine regulations no longer required it. This suggests a disregard for the person’s autonomy and inherent rights. Furthermore, NGO employees suggested that ethical considerations were not taken into account during the decision-making process. Instead, political considerations, such as the potential reaction of the population, took precedence.

“It is always bad, the pandemic has made it even worse […] it was no longer humane, it is already not humane” [NGO employee, m].

### Expectations for better support

Respondents identified several areas for improvement and assumed that the pandemic would have been less disruptive with greater support available. This applies to everyday, structural, and overarching measures. Concerning everyday measures, respondents indicated that to facilitate effective communication, the installation of multiple Wi-Fi routers would have helped. Equally significant would have been the provision of internet access on mobile devices, offering refugees opportunities for social interaction and leisure-time activities. Maintaining access for volunteers within the facilities has unanimous support to alleviate refugees’ anxieties and maintain human relationships according to the interviewees. Directors of shelters and NGO employees considered improved communication with refugees as fundamental factors to counter misinformation. In addition, NGO workers considered individualized care during quarantine as an essential measure to address the diverse physical and psychological needs of refugees.

Regarding structural measures, all stakeholders considered the use of native-speaking mediators as an important step to build trust with refugees. To promote the dissemination of information, NGO employees emphasize the importance of training for social workers within the facilities. Clear guidelines and instructions need to exist to ensure structure and safety in quarantine facilities, as suggested by NGO employees. Furthermore, interviewees considered it crucial to provide essential resources such as disinfectants and masks for the health and safety of refugees and staff. They further emphasized the importance of ensuring consistent implementation of measures to avoid confusion and ensure predictability. All interviewees requested better health education on COVID-19 provided by health authorities to shelter staff, NGO employees and refugees. This would provide all stakeholders with accurate information and prevention strategies. The promotion of integration and the acquisition of language skills were regarded to be an important factor as well. Furthermore, respondents noted the need to include the vulnerability of refugees in medical assessments in order to provide more tailored and effective support.

The proposed measures aim to address the concerns and recommendations of refugees, NGO employees, and refugee shelter managers to optimize assistance for refugees. A key demand was the establishment of an ethics committee to review and approve interventions and intervene in exceptional situations if necessary. NGO employees recommended the involvement of different parties from different institutions in decision-making processes regarding the provision of assistance to refugees. This would ensure a better assessment of the situation and the feasibility of interventions in institutions. NGO employees also called for more information dissemination at the political level in order to promote transparency and improve public understanding of measures to support refugees.

## Discussion

Despite the global attention given to pandemic-related restrictions, it appears that the scientific literature may not have fully explored specific circumstances of those living and working in confined settings such as refugee shelters. This investigation examined the perspectives of refugees, NGO employees, and directors on COVID-19 measures in German refugee shelters. It provides insights into their experiences with the measures and suggestions for improvement to better prepare for future pandemics.

The results highlight the overall difficult situation in refugee shelters during the COVID-19 pandemic and illustrate the challenges faced also by NGO employees and shelter directors. The measures implemented in the facilities we interviewed resemble those mentioned in previous studies [18, 21]. Compared to the measures recommended in guidelines and other studies, the measures taken to deal with the pandemic in the shelters appeared to be more improvised and without consideration of the impact on the mental and physical health of those involved [13]. The measures taken are in contradiction with EU-wide legal norms that provide for special care and protection of refugees. These standards aim to ensure an adequate standard of living, adequate housing and access to health care [5, 28, 29]. A further focus lies on the guarantee of the individual right to prevent and combat epidemics, which, according to those interviewed in this study, was not granted to the refugees.

Our findings suggest that the pre-existing psychological distress of refugees and their insecure residence status were exacerbated by the quarantine measures, especially among refugees who were subject to collective quarantine. These findings are also in line with the results of other studies in which refugees reported increased psychological distress as a result of quarantine [21, 30, 31]. In some shelters, the collective quarantine was ordered despite the fact that it has no epidemiological advantage over other measures. During the COVID-19 pandemic, refugees were highly dependent on external support, which was largely provided by local NGO employees, especially during the quarantine period. The interviews also showed that employees of NGOs took responsibility for communicating and informing refugees in many places. Additionally, they coordinated communication between refugees, authorities, and doctors. This emphasizes the dedication and importance of NGO employees. In our study, NGO employees and facility managers suggested to place refugees faster in their own homes, instead of providing housing in shelters for months or even years. This recommendation is not only supported by the institutions concerned, but also by other research [5, 13, 32]. They underline the effectiveness of this measure as a possible way to address the challenges posed by the specific spatial and health conditions of refugee accommodations. Faster integration into their own homes could not only address current administrative challenges, but also improve the physical and mental health of refugees. Other studies emphasize the importance of intersectoral work with various actors for effective crisis management. Research on pandemic measures in refugee accommodations shows that intersectoral collaboration can be essential to support measures in shelters and to secure necessary resources, such as protective materials and shelter or quarantine capacity [18].

Our results show that a development of pandemic plans for refugee shelters is needed and could serve as a guideline for future public health crises. This recommendation is also supported by insights from other studies conducted in this context [13, 21, 22, 33]. It is crucial to not only rely on the expertise of epidemiologists and other experts but also to involve representatives of the refugee community and an ethics commission in the development process. This ensures that the conceptual development incorporates not only scientific expertise but also addresses the needs and experiences of those affected. There is a high demand for both scientific and ethical evaluation of the implemented measures as well as practical expertise in the field of infection epidemiology to ensure a comprehensive and effective pandemic plan.

Some limitations of the present study need to be considered. Only one of the interviewed refugees was female. Furthermore, we were only able to conduct interviews with refugees in three languages. This may not adequately cover the perspectives of other language groups among the inhabitants of refugee shelters. The insights from the present study should therefore be complemented by further investigations. It is important to acknowledge that perspectives on the measures taken during the course of the pandemic and changing living conditions evolve dynamically. Therefore, our results can neither be generalized to all refugees and all refugee shelters nor to all phases of the pandemic.

## Conclusion

In conclusion, our investigation sheds light on the overlooked challenges faced by those living and working in confined settings, particularly in German refugee shelters, during the COVID-19 pandemic. Despite the global focus on pandemic-related restrictions, our study reveals that the scientific literature may not have fully explored the specific circumstances of this vulnerable population. The experiences and perspectives gathered from refugees, NGO employees, and shelter management highlight the overall difficult situation within refugee shelters, exposing challenges faced by all parties involved. Implemented measures appear to have been more improvised, lacking consideration for the mental and physical health of individuals. Our findings suggest that quarantine measures exacerbated the pre-existing psychological distress of refugees, especially those subjected to collective quarantine. NGO employees emerged as crucial support during the pandemic, assuming responsibility for communication and coordination between refugees, authorities, and healthcare providers. Our findings indicate a general consensus among stakeholders for transitioning refugees to independent housing, given the critical views on camp-like accommodations and their long-term viability. Stakeholders highlighted the significant mental and physical health risks associated with camp-like accommodations, which are corroborated by broader evidence showing negative health implications even outside of pandemic times. In terms of current accommodation settings, our study emphasizes the necessity for developing pandemic plans specifically tailored for refugee shelters. These plans should be seen as an interim measure to improve conditions in the short term, while efforts to transition to more suitable housing options must continue. To ensure these pandemic plans are effective and comprehensive, they should involve not only epidemiologists and experts but also representatives of the refugee community and an ethics commission. This inclusive approach would address both scientific and ethical considerations, ensuring that the measures implemented are respectful of the refugees’ dignity and well-being.

## Electronic supplementary material

Below is the link to the electronic supplementary material.

Supplementary Material 1

## Data Availability

Data are available from the authors upon reasonable request.
