# Supplementary material for: “The police came in white protective suits and with batons, it was pure disaster” – a multi-stakeholder perspective on infection control in reception centers for asylum seekers during the COVID-19 pandemic in Germany
Source: BMC Public Health. 2024 Sep 9;24:2445. doi: 10.1186/s12889-024-19925-5 (PMC11382394; doi:10.1186/s12889-024-19925-5)
Supplement: Supplementary file 1 — Supplementary Material 1 [file 12889_2024_19925_MOESM1_ESM.docx]

**Supplement for:**

***“The police came in white protective suits and with batons, it was pure disaster” – A multi-stakeholder perspective on infection control in reception centers for asylum seekers during the COVID-19 pandemic in Germany***

**Interview guides**

**Guide for narrative interviews with refugees in refugee shelters**

1. **Opening question/narrative prompt**

I am interested in hearing about your experience during the pandemic in the initial reception facility. Please tell me how the pandemic started and how it changed your life in the accommodation.

(Note to interviewer: During the narration of the conversation partners, strive for a conversation as non-directive as possible, i.e., no probing questions or interruptions, but active listening)

1. **Follow-up questions**

Picking up on open narrative threads with additional narrative prompts.

For example:

"You mentioned that there were contact restrictions in your accommodation. How did this affect your social contacts? Were you still able to meet friends or engage in your usual leisure activities?"

"During the quarantine period, how did you spend your days? What did you do all day? How did you feel about it?"

"When counseling centers closed, did you still have access to counseling? How did you inform yourself about the asylum procedure (or other topics)?"

"When the social welfare office was closed, how did you get your treatment vouchers during that time?"

etc.

1. **Evaluation phase**

"Now that you have shared how you experienced the pandemic: What, in your view, could have been done better?"

"Is there anything else you would like to tell me?"

**Guideline for interviews with NGOs responsible for refugees in refugee shelters**

| ***Measures developed to deal with the pandemic*** |
| --- |
| - When did the shelters under your responsibility start preparing for the pandemic? - Who initiated the measures? - Which measures were taken first? - What measures were then taken later on? - What influence did the COVID-19 pandemic have on the work in the refugee shelters? |
| - To what extent have the measures to contain the pandemic been followed by the refugees? - Which of the measures taken during the pandemic will remain in place in the future? |
| ***Shelter-related challenges*** |
| - Do the shelters offer vaccinations, or can refugees be vaccinated against COVID-19 elsewhere? - To which extent were/are the refugees informed about the existing vaccination options? - If applicable: How is the offer accepted by the refugees? |
| ***Conflicts during the pandemic*** |
| There have been a number of reports in the media about conflicts in refugee shelters related to pandemic measures.   - Were there any such conflicts in the shelters due to the measures taken during the pandemic? (between refugees and employees in the facilities) |
| ***Ethical and social aspects of the measures*** |
| In connection with the pandemic there has been and still is a general debate revolving around the legal and ethical justification for the pandemic measures. However, the situation of refugees has not been discussed as intensively in this debate.   - How do you view the measures taken by the shelters under your responsibility in this context? Have the fundamental rights/freedoms of refugees been restricted by the measures taken during the pandemic? - In your view, what legal or ethical considerations are suitable to justify the encroachments on refugees' freedoms? |
| The topic of mental health during the pandemic has been receiving increasing attention in recent weeks, with more discussions focusing on how to address the burdens of the pandemic – particularly among children.   - Alongside the predominantly restrictive measures for refugees in the shelters under your jurisdiction during the pandemic, were there also measures implemented specifically addressing the needs of refugees or alleviating their burdens? For example, were there any initiatives aimed at bridging or coping better with the measures associated with the pandemic? |
| ***Wishes/expectations for better support in times of pandemic*** |
| If you had the opportunity, what would you wish for to support your work with refugees during the COVID-19 pandemic?   - What can be learned from the pandemic for the future? |

**Guideline for interviews with social welfare offices responsible for refugees in refugee shelters**

| ***Measures developed to deal with the pandemic*** |
| --- |
| - When did the shelters under your responsibility start preparing for the pandemic? - Who initiated the measures? - Which measures were taken first? - What measures were then taken later on? - What influence did the COVID-19 pandemic have on the work in the refugee shelters? |
| ***Shelter-related challenges*** |
| - Do the facilities offer vaccinations, or can refugees be vaccinated against COVID-19 elsewhere? - How is the assumption of costs for COVID-19 vaccinations organized? - To which extent were/are the refugees informed about the existing vaccination options? - If applicable: How is the offer accepted by the refugees? |
| ***Ethical and social aspects of the measures*** |
| - Was there additional funding in your district to implement such measures to provide relief and psychosocial support for the refugees? If so, where did these funds come from? |
| ***Wishes/expectations for better support in times of pandemic*** |
| Finally, I would like to invite you to draw some conclusion about the pandemic and its impact on everyday life in the facilities under your responsibility.  - What do you think could have improved care in the facilities?  - What can be learned from the pandemic for the future? |
